# Recent Trends in the Management of Eosinophilic Esophagitis: A Systematic Review

**DOI:** 10.7759/cureus.43221

**Published:** 2023-08-09

**Authors:** Priyata Dutta, Prince Shah-Riar, Sumaita Sadida Bushra, Sharar Naiarin Haque, Zahin Islam Rafa, Fadi Hawa, Swarna Chakrabarty, Supti Dev Nath, Humayra Afrin, Nishat Shama, Farzana Khair, Sadia Maisha, Progga Kapuria, Barna Dam

**Affiliations:** 1 Internal Medicine, Trinity Health Ann Arbor, Ypsilanti, USA; 2 Internal Medicine, Ibn Sina Medical College, Dhaka, BGD; 3 Internal Medicine, University Muslim Medical Association Community Clinic, Los Angeles, USA; 4 Internal Medicine, Jahurul Islam Medical College & Hospital, Kishoreganj, BGD; 5 Internal Medicine, Dhaka Medical College Hospital, Dhaka, BGD; 6 Internal Medicine, Ibn Sina Medical College and Hospital, Dhaka, BGD; 7 Internal Medicine, St. Joseph Mercy Ann Arbor Hospital, Ann Arbor, USA; 8 Internal Medicine/Gastroenterology and Hepatology, University of Michigan, Ann Arbor, USA; 9 Internal Medicine, North East Medical College, Sylhet, BGD; 10 Pulmonary and Critical Care Medicine, Johns Hopkins University, Baltimore, USA; 11 Radiology, Mayo Clinic, Rochester, USA; 12 Internal Medicine, Bangladesh Institute of Research and Rehabilitation in Diabetes, Endocrine and Metabolic Disorders, Dhaka, BGD; 13 Internal Medicine, Bangladesh Medical College & Hospital, Dhaka, BGD; 14 Internal Medicine, Sher-e-Bangla Medical College, Barisal, BGD; 15 Internal Medicine, Z.H. Sikder Women's Medical College and Hospital, Dhaka, BGD; 16 Internal Medicine, Kumudini Women’s Medical College, Tangail, BGD

**Keywords:** eosinophilic esophagitis, protein pump inhibitors (ppi), elimination diet, eoe, eoe diet

## Abstract

Eosinophilic esophagitis (EoE) is a chronic immune-mediated condition characterized by inflammation and eosinophilic accumulation of the esophagus, resulting in dysphagia and food impaction. While the exact etiology of EoE remains unclear, it is believed to be triggered by food allergens and dynamic environmental factors, resulting in various clinical manifestations, from inflammation to fibrosis. Although clinical presentation varies with age, the number of eosinophils in esophagogastroduodenal endoscopy remains the diagnostic gold standard. While diet elimination, proton pump inhibitors (PPIs), topical corticosteroids, and biological therapy are promising treatment options for EoE, there are insufficient data to determine the optimal therapeutic treatment approach. Combination therapies - the use of dietary therapies in conjunction with other treatment modalities, such as PPIs, topical corticosteroids, or biologic agents - have also emerged as a potential management strategy for EoE. In this systematic review, we attempt to highlight the recent advances in EoE therapies and provide updated guidance to their management. From 2017 to 2022, we conducted a comprehensive electronic search of PubMed (MEDLINE) using specific keywords related to our objective and eventually included a total of 44 articles.

## Introduction and background

Eosinophilic esophagitis is a sporadic, long-standing, T helper type 2 (Th2) immune-mediated clinicopathologic condition characterized by eosinophil-predominant esophageal inflammation and limited to the esophagus [1,2]. Over the past two decades, EOE has become a significant cause of chronic gastrointestinal morbidity [3]. Although EOE is classified as a rare disease, its incidence and prevalence have increased significantly in recent years [4]. An estimated five to 10 new cases per 100,000 people per year are reported worldwide [5].

In the USA, the prevalence is up to 150 000 cases, with approximately one in 2000 people [4]. Histologically, EOE is detectable by a dense epithelial eosinophilic infiltrate [6]. Endoscopic mucosal biopsy findings of >15 eosinophils per high-power field highly suggest EOE. The symptoms of EOE include dysphagia, heartburn, food impaction, and regurgitation. However, upper gastrointestinal (GI) endoscopy is the gold standard for evaluating patients with EOE. Amino-acid-based elemental diets establish EoE as a selective form of food allergy. An empiric six-food elimination diet (SFED) induces histological remission in adults with active EoE. Six-food elimination diets include cow’s milk, eggs, soy, wheat, peanuts/tree nuts, and fish/shellfish, which have been extensively used. In the past, proton pump inhibitors (PPIs) and topical corticosteroids were thought to be the most effective treatments for EOE; however, recent studies show that a combination of proton pump therapy and a food elimination diet can reduce the severity and progression of EoE [7].

In this systematic review, we aim to examine the recent advances in available treatments of EOE and provide updated guidance for its management.

## Review

Methodology

We systematically reviewed the articles published in the last five years by following Preferred Reporting Items for Systematic Reviews and Meta-Analyses (PRISMA) guidelines [8]. We conducted a comprehensive online search strategy for data collection using MEDLINE (accessed from PubMed). Our search terms include but are not limited to “EoE”, “diet, elimination diet, Six Food Elimination Diet (SFED), Four Food Elimination Diet (FFED), Two Food Elimination Diet (TFED), One Food Elimination Diet (OFED), skin prick test, allergy test”.

Inclusion criteria were as follows: 1) Research articles, such as both narrative and systematic reviews and meta-analyses, cross-sectional studies, and longitudinal studies, were included, 2) Studies focusing on the adult population, 3) Studies comparing two or three EoE modalities. Exclusion criteria included pediatric cases (age <18 years), studies written in foreign languages, animal and in-vitro studies, case reports, and case series.

**Figure 1:**
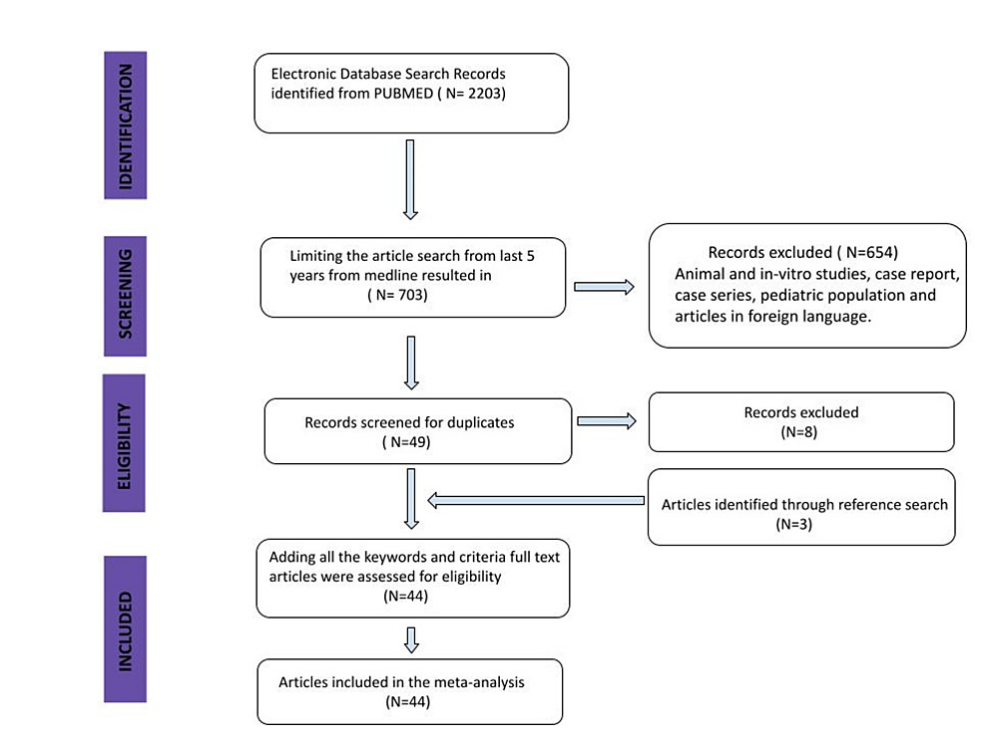
PRISMA flow diagram of the included studies PRISMA: Preferred Reporting Items for Systematic Reviews and Meta-Analyses Author's own creation

A total of 703 articles relevant to our initial keywords were retrieved from the online database. After extensive screening and full-text reviewing, we selected 44 articles (Figure 1). For four months, all the articles went through extensive deliberations, including scanning for relevant information and paraphrasing the essential points for analysis in data spreadsheets. Any discrepancies were solved among the authors through extensive discussion. Key findings of previously published studies are summarized in Table 1.

Pathophysiology

Eosinophilic esophagitis is a multifaceted disorder caused by a combination of genetic predisposition, host immunological response, and environmental triggers such as food. Dietary antigens primarily cause it; however, it can be exacerbated by aeroallergens such as pollen or spores, which can worsen the clinical picture without requiring significant dietary changes [9,10].

These antigens trigger the esophageal epithelium to express interleukin-33, thymic stromal lymphopoietin (TSLP), ultimately stimulating Th2 cells to release cytokines. In the long run, these cumulative inflammatory reactions reduce the epithelial barrier’s integrity, damage the mucosa, and cause esophageal fibrosis. Underlying genetic structure, previous atopic disorders, and exposures in early life can precipitate the likelihood of EoE development [11]. In addition, food products are primarily responsible for allergic reactions in EoE. This is evidenced by the remission of histological and clinical features with food and the worsening of symptoms with food reintroduction. However, the inadequate manifestation of an immediate reaction to food products, the less predictive value of SPTs (skin prick tests), and the insufficient improvement with immunoglobulin E (IgE) therapy invalidate the speculation that EoE is solely an IgE-mediated food reaction, similar to the pathogenesis of atopic disorders such as asthma and eczema [12]. Therefore, the existence of asthma, atopic dermatitis, and IgE-mediated food allergies is collectively related to the later diagnosis of EoE [13]. The natural history of EoE depicts a gradual progression from the inflammatory stage to the fibrostenotic change, with esophageal dysmotility potentially resulting from esophageal remodeling [10].

Diagnosis of EoE

The diagnosis of EoE requires both clinical symptoms and endoscopic as well as histologic findings.

In 2007, the American College of Gastroenterology (ACG) established the following diagnostic criteria for esophageal dysfunction: 1) the presence of symptoms related to esophageal dysfunction; 2) esophageal biopsies revealing an abundance of eosinophilic inflammation (usually 15 or more eosinophils per high-power field); 3) eosinophilia that is limited to the esophagus and persists despite treatment with PPIs; 4) according to the criteria, it is necessary to eliminate other possible factors that could cause esophageal eosinophilia.; 5) the patient’s response to treatment with dietary changes and topical corticosteroids, although this response is not necessary for diagnosis [14]. However, later in 2011, these diagnostic criteria were updated as follows: 1) clinical manifestation of esophageal dysfunction; 2) isolated esophageal eosinophilic infiltration (15 or more eosinophils per high power field); 3) diagnosis of potential non-EoE conditions that can cause esophageal eosinophilia [15].

Early diagnosis of EoE is crucial, as EoE progresses to fibrostenosis quickly if it remains untreated. Moreover, there is a 9% increase in stricture formation annually with undiagnosed EoE [16]. Esophageal dysfunction is characterized by the persistent presence of the following symptoms: dysphagia, food impaction, heartburn, food refusal, regurgitation, vomiting, and chest pain. In young patients with allergic conditions, typical symptoms, such as solid food dysphagia or recurrent food bolus impactions, increase the pretest probability of EoE [17]. The second step of diagnosis is an endoscopic esophageal biopsy showing ≥15 eosinophils per high-power field (HPF). The ACG recommends obtaining a minimum of six biopsies in a suspected EoE patient [11,18].

Linear furrows, mucosal rings, small diameter esophagus, white plaques or exudates, edema, long- and short-segment stricture Schatzki rings, corrugated rings, and crepe-paper mucosa (e.g. esophageal mucosal tearing from the passage of an endoscope) are the most prevalent endoscopic findings in adults with EoE [1,19]. Increased intraepithelial eosinophils in the esophagus without concurrent eosinophilic infiltration in the stomach or duodenum continue to be considered the gold standard for EoE diagnosis in cases where upper gastrointestinal endoscopy has limited sensitivity for detecting accurately fixed luminal constriction [20,21].

The next step is the exclusion of other causes of esophageal eosinophilia such as gastroesophageal reflux disease, achalasia, eosinophilic gastrointestinal disease, hypereosinophilic syndrome, connective tissue diseases, Crohn’s disease, infections, pill-induced esophagitis, and graft versus host disease. Therefore, EoE diagnosis can take an average of up to six years, as the symptoms of EoE are nonspecific, necessitating a thorough medical history [22].

Goal of treatment

The goal of EoE treatment can be divided into two parts: a short-term goal and a long-term goal. The treatment’s short-term goal is to reduce patients’ symptoms, which is one of the diagnostic criteria, and to improve histological findings by lowering eosinophil counts on biopsy [17]. As EoE is a chronic disease, the long-term goal of treatment is to prevent disease progression and complications (e.g. esophageal stenosis) and disease recurrence [19].

**Figure 2:**
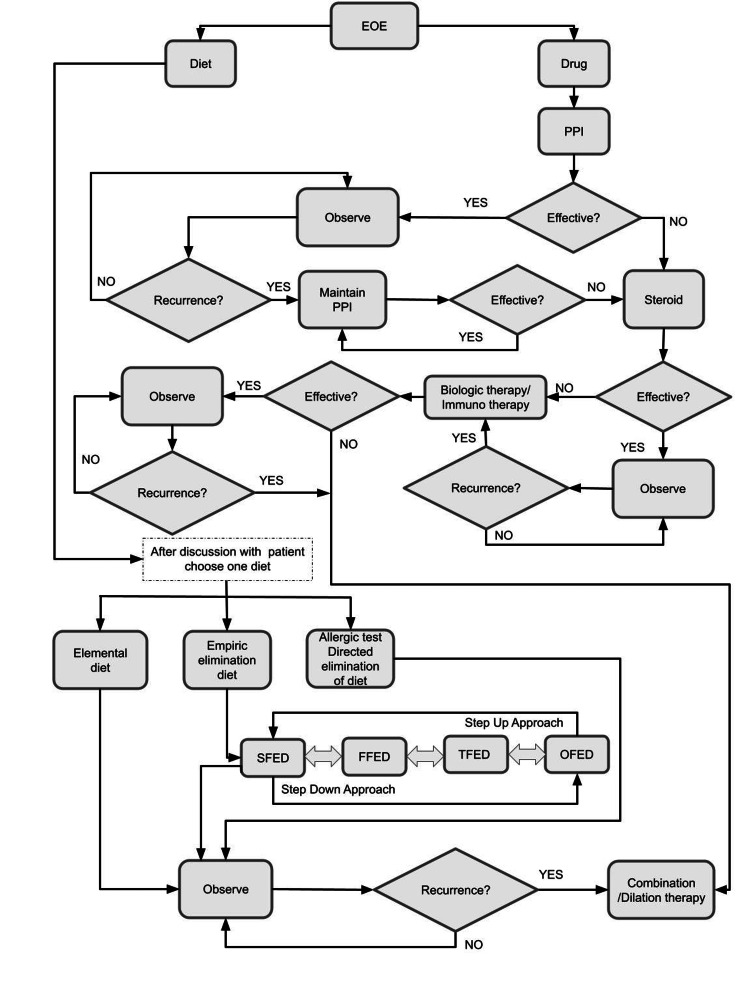
Treatment algorithm for eosinophilic esophagitis Author's own creation EoE = Eosinophilic Esophagitis, PPI = Proton Pump Inhibitor, SFED = Six-Food Elimination Diet, FFED = Four-Food Elimination Diet, TFED = Target-Food Elimination Diet, OFED = One-Food Elimination Diet

Treatment options for EoE can be dietary therapy, pharmacological therapy, or combination therapy, depending on the patient’s choice and clinical course (Figure 2). PPIs, corticosteroids, allergen immunotherapy, and biologic therapy are all pharmacologic options. Emerging biologic therapy is creating a new era for EoE management. Esophageal dilation could be a good treatment option for complicated EOE (e.g., patients who have already developed esophageal stenosis) management in combination with corticosteroids. [23,24]. The current standard of care is to begin PPI in suspected EoE patients, and steroids and dietary therapy can be considered if PPI is ineffective [14].

When single pharmacotherapy or dietary therapy fails to achieve histologic remission, combining a food-elimination diet with PPIs can achieve histological remission. Even though there are few studies on combination therapy, it can be a viable option for those who have failed monotherapy [7].

Dietary approaches

Eosinophilic esophagitis is a non-IgE-mediated allergic disease mainly induced by food proteins. It has been demonstrated that avoiding contact between the esophageal surface and food proteins reliably resolves symptoms and inflammation [1].

The dietary approach requires a strong-willed patient, a motivated physician, and the availability of nutritional resources. Dietary therapy can be effective for long-term EoE treatment [19].

The goal of the diet is to determine which foods cause EoE in each patient. A diet plan that only prohibits the triggering items can be created using this procedure to meet the needs of a specific individual [25].

Empiric Elimination Diet

**Figure 3:**
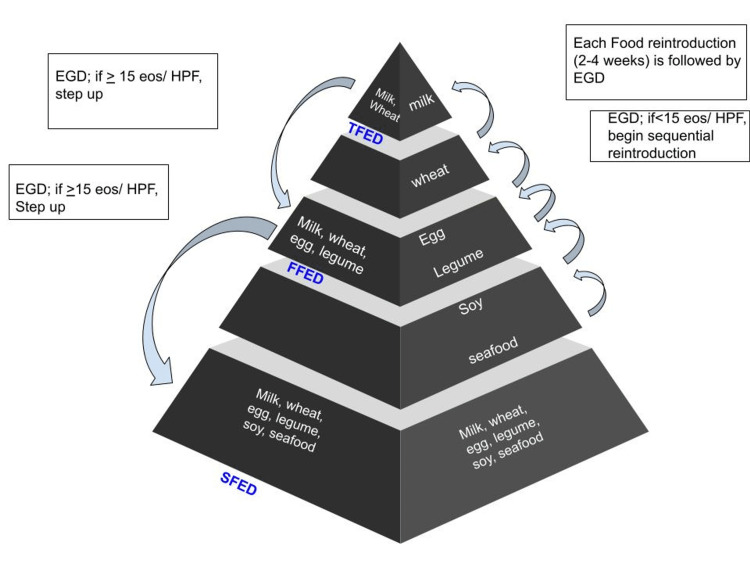
Empirical Diet Therapy Author's own creation EGD = Esophagogastroduodenoscopy, EoS = Eosinophil, TFED = Two-Food Elimination Diet, FFED = Four-Food Elimination Diet, SFED = Six-Food Elimination Diet [26-31]

The empirical elimination diet, known as SFED, is one of the most popular diet approaches to treating EoE. Usually, empiric elimination diets last six to eight weeks, followed by a repeat endoscopy - an empiric elimination diet based on the patient’s allergic response to a particular food. Currently, the best evaluated dietary treatment modality in EoE is elimination followed by stepwise and controlled reintroduction (step-down strategy); the six most critical food categories are SFED - milk, gluten, egg, soy, peanuts/tree nuts, fish, and seafood [1,20,26]. Reintroduction is always maintained in a controlled environment, as it requires serial endoscopies, which are both cost- and time-intensive, and is considered a drawback. For this reason, 1- or 2-FED (one/two-food elimination diets) followed by a step-up strategy is increasingly advocated (Figure 3) [27].

Endoscopic biopsy is usually performed to check for the histologic response after the elimination of a food group when patients demonstrate a histologic response to monitor whether the disease will reoccur after the sequential reintroduction of that food group. Though there are no standardized approaches to food reintroduction and follow-up endoscopy, endoscopy is usually performed after reintroducing one to two foods [1,20,28]. 

Depending on how many foods were initially eliminated, a common technique is to introduce two or more foods sequentially at two-week intervals before having regular endoscopies. For instance, a patient on an SFED might start with seafood for two weeks; if the patient is asymptomatic, they might then introduce peanuts and tree nuts for two weeks, followed by an endoscopy to determine how these two food groups affect their histology. Less allergenic foods with lower protein contents, such as fruits and vegetables, are typically introduced to patients who have undergone more thorough elimination diets before more allergenic foods [10,29].

Eliminating even a few widely consumed meals could be challenging and have an impact on a patient’s quality of life (QOL) in the long run. Concerns about dietary contamination, the psychosocial effects of dietary restrictions, and the cost of allergen-free foods can make the elimination diet difficult to implement in practice. The effects of EoE on social interactions and food were of concern to the patient. Moreover, SFED may further harm patients’ QOL by purposefully restricting their diets. Thus, the success of the elimination diet method is probably enhanced by bringing in a dietician and an allergist to provide patient education and dietary monitoring. The process of diet reintroduction may also be more enjoyable due to less invasive technologies for esophageal sampling [20,30-31].

Elemental Diet

Elemental diet therapy consists solely of amino acid-based formulas without solid food intake. However, there are currently two possible indications: patients who are resistant to medical and less restrictive dietary treatments and a radical initial modality to induce remission in patients with severe illness [1].

Though amino-acidic-based diets have impressive remission rates, they can hamper everyday life.

One-Food Elimination Diet

Multiple studies have assessed the efficacy of OFED, in which only milk or wheat/gluten was restricted. For example, 34 studies with 1762 patients showed histologic remission on OFED of 51.4.%, whereas the overall rate of clinical response was 87.1% [32].

Four-Food Elimination Diet

The FFED aims to avoid unnecessary endoscopies and make the diet more tolerable for patients. Dairy, wheat, eggs, and legumes are restricted. Multiple studies show that the rate of histologic remission on FFED is almost twice that of the placebo group. According to Rooji et al., adding FFED to an amino acid formula does not significantly reduce peak eosinophil count compared with OFED but significantly improves QOL [33,34].

Target Elimination Diet

The most effective and elegant treatment for EoE is identifying and subsequently eliminating the culprit food group(s) [1]. Target elimination diets aim to prevent empirical eliminations by identifying and limiting specific trigger foods based on the results of food allergy tests such as skin prick tests and atopy patch Tests. Skin prick tests (SPTs) and atopy patch tests (APTs) have also been investigated in this context; Patients without immediate food reactions may not experience predictable results with SPT while APT may not be a dependable method for identifying food triggers in those with EoE. As opposed to APTs, which measure delayed non-IgE, cell-mediated reactions, SPTs reveal immediate IgE-mediated allergic reactions [9].

Skin Allergy Testing-Directed Elimination Diet

Different types of allergy testing, such as skin prick tests, atopy patch tests, serum IgE, and microarrays, can be done to determine the triggering food group(s). Though the testing rate is disappointingly low, as those tests are based on IgE, IgE only partially plays a role in EoE [35]. Allergy test-directed diets result in lower remission percentages due to their poor ability to identify the offending foods [19].

SFED: The “Classic” Top-Down Approach

In this approach, patients first eliminate a large group of foods from their diet for at least six weeks. These foods include milk, wheat, eggs, soy, nuts, and seafood. After this initial period, the foods are gradually reintroduced one at a time. This allows the identification of specific food triggers that are causing allergic reactions. The process of food reintroduction is also evaluated histologically [32].

Angelika et al. discovered that the overall histologic response for SFED was 54%, with a single food trigger identified for most individuals following reintroduction [34].

The Step-Up Approach

Extensive dietary restrictions and endoscopic procedures are believed to be the most effective deterrents for patients and physicians in elimination diets. Since most EoE patients only have one or two trigger foods, not all therapeutic-diagnostic testing may be required [25].

**Figure 4:**
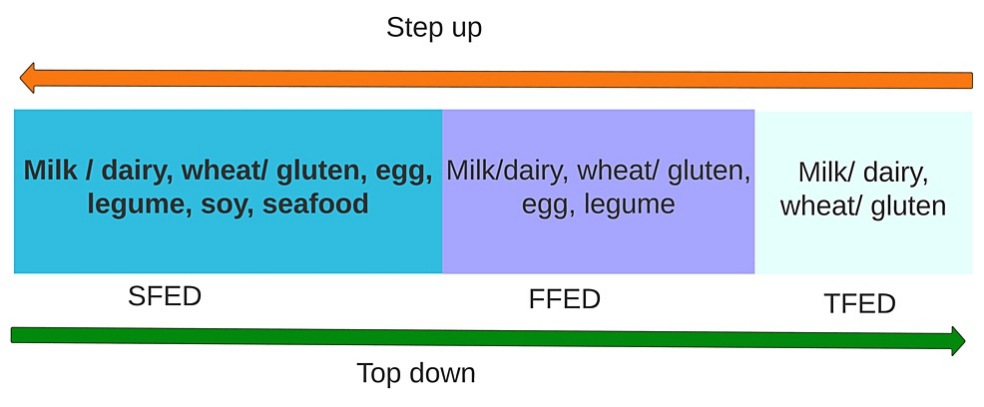
Step-up and top-down approach Author's own creation SFED: Six-Food Elimination Diet, FFED: Four-Food Elimination Diet, TFED: Three-Food Elimination Diet

This approach begins with TFED. Then, if patients do not have enough response, it is stepped up to FFED and, subsequently, SFED. However, 6-FED (milk, wheat, egg, soy/legumes, nuts, fish/seafood) is considered the standard dietary therapy in clinical practice (Figure 4) [2,35,36].

PPI + histologic and symptom response 

Treatment should begin with a once-daily, full-dose PPI, and symptomatic response should be evaluated after eight weeks. However, if symptoms persist after four weeks, the PPI dose can be increased to twice daily [22]. If a patient responds to this treatment, PPI should be continued at the lowest daily dose [18]. Furthermore, EoE can cause reflux symptoms through two mechanisms: first, impairment of the lower esophageal sphincter, which can result in secondary reflux, and second, hypersensitivity of the eosinophil-inflamed esophageal mucosa, which can result in reflux-like symptoms even in the absence of true reflux [1]. Several studies on EOE treatment found evidence of PPI-responsive and PPI-resistant EoE with different pathophysiologies.

In EoE patients, Th2 cytokines in esophageal epithelial cells secrete the pro-inflammatory chemokine eotaxin-3. Omeprazole directly inhibits epithelial signal transducer and activator of transcription 6 (STAT6), which downregulates the secretion of eotaxin-3. However, despite continuous PPI therapy, patients with allergic rhinitis and cytochrome P450 2C19 (CYP2C19) rapid metabolizers are at higher risk of developing PPI-resistant EoE [13,37]. PPIs also demonstrate anti-cytokine effects on the esophageal epithelium [38].

A therapeutic regimen of PPI is also used to differentiate between PPI-responsive and PPI-resistant EoE. Patients are given an eight-week trial of PPI followed by an endoscopic mucosal biopsy. In PPI-resistant EoE, persistent eosinophilia is defined by more than 15 eosinophils per high-power field [38,39]. However, some researchers suggest that the PPI trial should be kept in the study because it may lessen the number of endoscopies needed, help with concurrent GERD, and offer a step-by-step method for diagnosing EoE. In conjunction with the use of a PPI trial, the ability to discuss a variety of medications (such as those used for classic EoE) without initially committing patients to a PPI was deemed advantageous [15].

Glucocorticosteroids

Glucocorticosteroids play a vital role in the medical treatment of EoE; however, they have some limitations, as they are not a permanent cure because they can only slow the progression of the disease. For instance, the European Medicines Agency (EMA) has successfully approved the tablet formulation of budesonide, whereas, in the USA, no studies have been approved by the Food and Drug Administration (FDA) [1]. Commonly prescribed glucocorticoids are budesonide, fluticasone propionate, and ciclesonide. A clinical trial of 88 patients with EOE showed 58% complete remission in six weeks with oral budesonide compared to no remission in the placebo group [14].

In another study of 229 patients, administration of topical corticoids resulted in higher rates of clinical remission (31.0% vs. 4.5%), histological remission (44.8% vs. 10.1%), endoscopic remission (48.8% vs. 17.8%), and complete remission (16.1% vs. 1.3%) compared to the opposite cohort. In another clinical trial of a placebo-controlled group for 50 weeks, a twice-daily maintenance dosage of 0.25 mg of deglutenized budesonide was administered. Results showed a higher frequency of clinical and histological remission, coupled with remodeling events and apoptosis of epithelial cells. Non-eosinophilic markers of inflammation were also reduced, and the treatment was found to have no significant side effects [14].

However, the AGA and Joint Task Force (JTF) recommend topical glucocorticosteroids over systemic glucocorticosteroids for patients with EoE due to similar efficacy and fewer severe side effects [40,41].

Emerging biologic therapy

Eosinophil targeted therapy, particularly interleukin-5 (IL-5) and interleukin-13 (IL-13), has become an attractive option for EoE therapy due to its crucial role in proliferation and survival. Interleukin-5 is the primary mediator for the maturation of eosinophils, and IL-13 is the master regulator of EoE pathogenesis, the driving force of IL-5 production, and induces epithelial barrier dysfunction [9,42]. There is a biologically credible mechanism that supports the use of anti-IL-5 therapy in patients with EoE due to the role of IL-5 in the maturation and release of eosinophils; a study suggested that while we witnessed a decrease in tissue eosinophilia, very few of them achieved histologic remission with < 15 eosinophils/hpf [43]. Another study focusing on a broad EoE population and using mepolizumab or reslizumab or both, an alternative anti-IL-5 agent, repeatedly demonstrated a reduction in disease activity but not remission. Other eosinophil-targeted therapies, such as benralizumab and anti-siglec-8 biologics, have direct cytotoxic effects on eosinophils, which might be advantageous compared to therapies targeting the IL-5 cytokine. However, investigations into these therapies are still ongoing and might provide future therapeutic possibilities [44].

Despite having several IL-13-targeting agents under development, dupilumab is the only clinically approved anti-IL-13 agent available. According to a recently published study, dupilumab offers a promising prospect for EoE treatment due to its effectiveness against a broader aspect of atopic disease. Despite a brief trial period, dupilumab was well-tolerated with no increase in peripheral eosinophilia. However, the effects of dupilumab on the broad EoE population, including individuals with atopic comorbidities, remain unknown and need further study [41]. Overall, there is low certainty in the effects of anti-IL-5 and IL-13 treatments on EoE management [38]. Alternatively, purine analogs (thiopurine, an immunosuppressant) demonstrated promising results in another study in a small group of adult patients with refractory EoE. However, this treatment option has not been further evaluated [1].

Esophageal dilation

Adult EoE patients with strictures may undergo esophageal dilation, which provides long-lasting symptomatic relief that can be provided by esophageal dilation but without showing any histological improvement [20]. Dellon et al. explored the role of endoscopic dilation in monitoring symptom response in the absence of histological response to diet or topical steroid therapy. The study found that dilation before or after these therapies could explain the symptom response in histological non-responders [24].

Combination therapy

There is very little data on the effectiveness of combination therapy, which includes PPIs, topical steroids, and dietary elimination. If multimodal therapy causes both symptomatic and histologic improvement, it is quite challenging to determine which modality is effective. Lower doses of a PPI or histamine H2-antagonist are required to control symptoms in patients with symptoms such as heartburn and acid regurgitation. Patients who do not respond to any first-line treatments should be evaluated for causes such as poor adherence, improper administration of topical steroids, inadequate dosing of medications, and fibrostenosis. However, it remains unknown whether multimodal therapy is as efficacious as single-agent therapy for refractory disease [11]. A retrospective study showed patients refractory to PPI or FED monotherapy may achieve symptom benefit and histologic remission successfully with PPI and FED combination therapy and may be considered first-line monotherapies [7]. They used omeprazole or esomeprazole as PPI and milk, soy, eggs, and wheat as FED. This study proved that combination therapy could be an effective tool in treatment strategies that fail as monotherapy [7].

Limitations

In this systematic review, all articles published before 2017 were excluded. The only search engine used was PubMed. Additionally, studies related to the pediatric population and animals were excluded. Our study only focused on the adult human population. Moreover, only articles written in the English language were selected. Therefore, our study sample was not fully representative of the general population.

## Conclusions

Dysphagia, food impaction, heartburn, regurgitation, vomiting, and chest discomfort are prevalent EoE symptoms. Although esophageal dysfunction symptoms, esophageal mucosal biopsies, and ruling out alternative esophageal eosinophilia sources are used to diagnose EoE, biopsy findings showing increased intraepithelial eosinophils are considered the gold standard for diagnosis. While comparing elimination diets as management options, such as OFED, TFED, FFED, and SFED, the ‘step-down approach’ reduces symptoms in a higher percentage of patients. Traditionally, PPI is used as a first-line drug for EoE treatment; however, glucocorticoids and newer eosinophil-targeted therapies are prescribed as an adjunct to PPI for unresponsive cases. Although PPI is commonly used as a standard treatment option, a combination approach to treatment can be a viable option for cases where monotherapy has been ineffective.
